# Staging right heart failure in patients with tricuspid regurgitation undergoing tricuspid surgery

**DOI:** 10.1093/ejcts/ezac290

**Published:** 2022-05-02

**Authors:** Xavier Galloo, Jan Stassen, Steele C Butcher, Maria Chiara Meucci, Marlieke F Dietz, Bart J A Mertens, Edgard A Prihadi, Pieter van der Bijl, Nina Ajmone Marsan, Jerry Braun, Jeroen J Bax, Victoria Delgado

**Affiliations:** 1 Department of Cardiology, Leiden University Medical Centre, Leiden, Netherlands; 2 Department of Cardiology, Vrije Universiteit Brussel (VUB), Universitair Ziekenhuis Brussel (UZ Brussel), Brussels, Belgium; 3 Department of Cardiology, Royal Perth Hospital, Perth, Australia; 4 Department of Cardiovascular and Thoracic Sciences, Fondazione Policlinico Universitario A. Gemelli IRCCS, Catholic University of the Sacred Heart, Rome, Italy; 5 Department of Bioinformatics, Bioinformatics Centre of Expertise, Leiden University Medical Centre, Leiden, Netherlands; 6 Hartcentrum, Ziekenhuis Netwerk Antwerpen (ZNA) Middelheim, Antwerp, Belgium; 7 Department of Cardio-Thoracic Surgery, Leiden University Medical Centre, Leiden, Netherlands; 8 Heart Centre, University of Turku and Turku University Hospital, Turku, Finland

**Keywords:** Prognosis, Tricuspid valve surgery, Right heart failure

## Abstract

**OBJECTIVES:**

This study evaluated the prognostic value of staging right heart failure in patients with significant tricuspid regurgitation (TR) undergoing tricuspid valve (TV) surgery.

**METHODS:**

Patients with significant TR who underwent TV surgery were divided into 4 right heart failure stages according to the presence of right ventricular (RV) dysfunction and clinical signs of right heart failure: stage 1 was defined as no RV dysfunction and no signs of right heart failure; stage 2 indicated RV dysfunction without signs of right heart failure; stage 3 included RV dysfunction and signs of right heart failure; and stage 4 was defined as RV dysfunction and refractory signs of right heart failure at rest.

**RESULTS:**

A total of 278 patients [mean age 64 (12), 49% males] were included, of whom 34 (12%) patients were classified as stages 1 and 2, 141 (51%) as stage 3 and 103 (37%) as stage 4 right heart failure. The majority of patients (91%) had TV surgery concomitant to left-sided valve surgery or coronary artery bypass grafting and 95% underwent TV annuloplasty. Cumulative survival rates were 89%, 78% and 61% at 1 month, 1 year and 5 years, respectively. Stages 1 and 2 and stage 3 were independently associated with better survival compared to stage 4 (hazard ratio: 0.391 [95% confidence interval: 0.186–0.823] and 0.548 [95% confidence interval: 0.369–0.813], respectively).

**CONCLUSIONS:**

Patients with significant TR undergoing TV surgery and diagnosed without advanced right heart failure have better survival as compared to patients with right heart failure.

**Figure:**
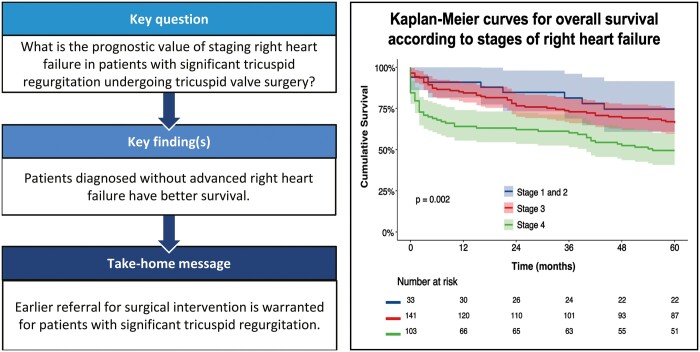

## INTRODUCTION

The prognostic implications of severe tricuspid regurgitation (TR) have been reported in several patient cohorts and left untreated, severe TR is associated with poor survival [1, 2]. Current guidelines advise tricuspid valve (TV) surgery as a concomitant procedure to left-sided valve intervention in patients with severe secondary TR or at an earlier phase in patients with mild to moderate secondary TR if dilatation of the tricuspid annulus (≥40 mm) or signs of right heart failure are present. Isolated TV surgery is recommended in symptomatic patients with severe secondary TR in the absence of severe right or left ventricular dysfunction or severe pulmonary hypertension [3, 4]. However, severe TR may remain asymptomatic for a long time and, when left untreated, severe TR leads to progressive right atrial and right ventricular (RV) dilation and dysfunction, eventually causing right heart failure symptoms and increasing the mortality risk of an intervention, regardless of left ventricular function and pulmonary hypertension [1, 5–7]. A recent staging system based on the presence of RV dysfunction and clinical signs of right heart failure has been associated with outcome in patients with severe TR who were largely treated medically [8–10]. This study evaluated whether a staging algorithm of right heart failure is associated with outcomes of patients with significant TR undergoing TV surgery.

## PATIENTS AND METHODS

### Study population

**Figure 1:**
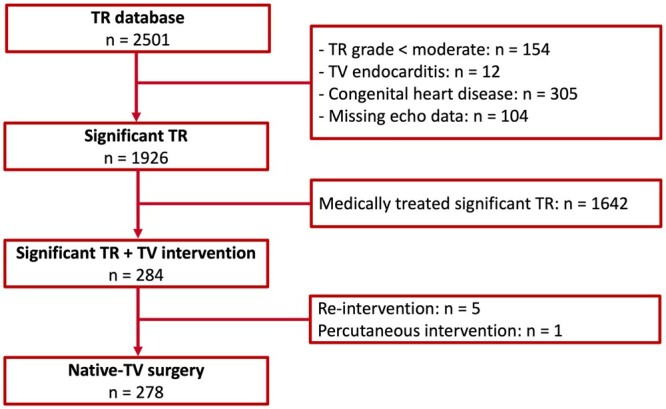
Flow chart for study population selection. TR: tricuspid regurgitation; TV: tricuspid valve.

Patients with significant TR who subsequently underwent TV surgery, after multidisciplinary discussion in the Heart Team applying the prevailing guidelines for surgical valvular intervention [3, 4], between January 2000 and September 2016 were identified from the departmental echocardiographic database of the Leiden University Medical Centre (Leiden, Netherlands). Significant TR was defined as moderate-to-severe TR [11]. Patients with primary or secondary TR, undergoing isolated or concomitant TV surgery as well as patients with a cardiac implantable electronic device were included in the current analysis. Patients aged <18 years or with prior TV surgery, active endocarditis, known congenital heart disease or with missing/incomplete echocardiographic data were excluded from the analysis (Fig. 1). Demographic and clinical data were retrospectively collected from the departmental Cardiology Information System (EPD-Vision, Leiden University Medical Centre, Leiden, Netherlands).

### Ethical statement

The institutional review board of the Leiden University Medical Centre approved the observational design and retrospective analysis of clinically acquired data (IRB: CME 10/053—18 May 2010) and waived the need for patient written informed consent.

### Clinical and echocardiographic variables

Baseline demographic, clinical, laboratory and echocardiographic variables were evaluated at the time of first diagnosis of significant TR on transthoracic echocardiography. Demographic characteristics included age, sex and body surface area. Clinical characteristics included symptoms of heart failure, relevant medical history and comorbidities and heart failure medication. Risk scores for predicting survival were calculated (with an in depth overview of the Tri-score, although currently only validated in patients undergoing isolated TV surgery, presented in Supplementary Material, Table S1 and Supplementary Material, Fig. S1).

Transthoracic echocardiograms were performed at rest using available equipment (Vivid 7, E9 and E95 systems, GE-Vingmed, Horten, Norway) and images were digitally stored for offline analysis (EchoPAC version 113.0.3, 202 and 203; GE-Vingmed, Horten, Norway). M-mode, 2-dimensional and colour and continuous- and pulsed-wave Doppler data were acquired from the parasternal, apical and subcostal views, according to current guidelines [11–13]. Left ventricular ejection fraction was quantified using the Simpson’s biplane method [12]. Left atrial volume was measured at end-systole on the apical four-chamber view and indexed for body surface area [12]. RV systolic function was evaluated by tricuspid annular plane systolic excursion (TAPSE), measured on M-mode recordings of the lateral tricuspid annulus on a focused RV apical view. TAPSE <17 mm was considered as RV dysfunction and used in staging right heart failure. Furthermore, RV end-systolic and end-diastolic areas were traced, and RV fractional area change was derived. Integrative assessment of valve function was performed through a multiparametric approach including qualitative, semi-quantitative and quantitative parameters measured on bidimensional, colour, continuous- and pulsed-wave Doppler data and was graded according to current recommendations [11, 13]. Systolic pulmonary artery pressure was estimated from the TR jet peak velocity applying the Bernoulli equation and adding right atrial pressure. Right atrial pressure was estimated based on the inferior vena cava diameter and its collapsibility during breathing (≤21 mm with >50% inspiratory collapse: 3 mmHg; >21 mm with ≤50% inspiratory collapse: 15 mmHg; intermediate situations: 8 mmHg) [12].

### Stages of right heart failure

**Figure 2:**
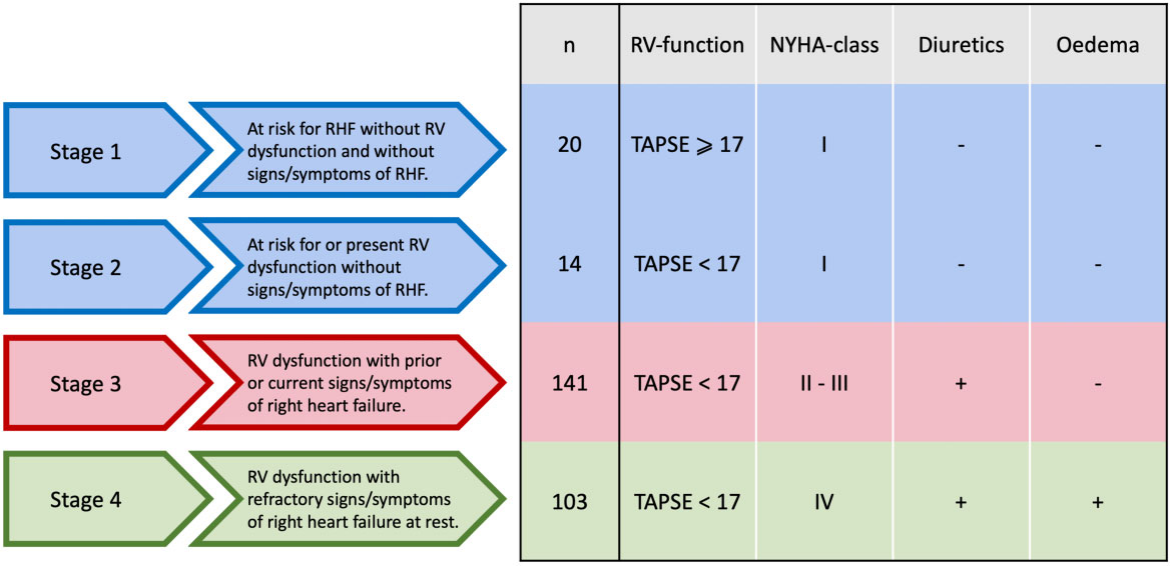
Stages of right heart failure defined by clinical and echocardiographic variables. NYHA functional class: New York Heart Association functional classification; RV: right ventricle; TAPSE: tricuspid annular plane systolic excursion.

Right heart failure was divided into 4 progressive stages of disease, as first proposed by Haddad *et al.* [8] and adapted by Dietz *et al.* [10] (Fig. 2). Patients categorized as stage 1 are at risk for right heart failure without RV dysfunction and without signs/symptoms of right heart failure. Patients in stage 2 had RV dysfunction without symptoms of right heart failure. Stage 3 includes patients with RV dysfunction and prior or current signs/symptoms of right heart failure, yet controlled with the use of diuretics, and stage 4 includes patients with RV dysfunction and refractory signs or symptoms at rest of right heart failure. Patients were classified according to the parameter that defined the highest stage. Stages 1 and 2 were combined for further statistical analysis, because of the low sample size in both stages.

### Follow-up and outcome definition

The study end-point was all-cause mortality. Outcomes were analysed from the time of surgery until death or the last follow-up in January 2021. Survival data were ascertained from the departmental Cardiology Information System and the Social Security Death Index. Peri- and postoperative morbidity and mortality rates were also analysed, according to the *European Journal of Cardio-Thoracic Surgery* guidelines [14]. Clinical follow-up was left at the discretion of the treating physician and data were collected by retrospectively reviewing the available information.

### Statistical analysis

Continuous variables with a Gaussian distribution are presented as mean and standard deviation and continuous variables without a Gaussian distribution are presented as median and interquartile range (IQR). Categorical variables are presented as frequencies and percentages.

Differences among the groups of right heart failure stage were analysed using the one-way ANOVA for continuous variables with normal distribution, the Kruskal–Wallis test for non-normally distributed continuous variables and the Pearson chi-squared test for categorical variables. Multiple comparisons for continuous variables were tested with the Bonferroni correction and *P*-value for trend analysis.

The Kaplan–Meier survival analysis was used to estimate the 5-year survival rate, and differences between groups were analysed using the log-rank test. Uni- and multivariable Cox proportional hazards regression analyses were performed to assess the clinical and echocardiographic factors that were independently associated with all-cause mortality. Variables that have been associated with outcome in valvular heart disease (age [15], sex [16], haemoglobin [17], chronic kidney disease [18], hepatic dysfunction [4], left ventricular ejection fraction [1], RV annular dilation [19], the presence of a RV lead [20], RV dysfunction [10] and concomitant mitral valve surgery [21]) were selected for multivariable regression analysis. In addition, for these variables, the number of missing values did not exceed 5% of the total study population and correlation factor analysis was used to determine if any pairs of variables were correlated. No collinearity (correlation coefficient of >0.60) was detected for the continuous variables that met the entry criteria for multivariable regression analysis. All *P*-values were two-sided, and values <0.05 were considered significant. All data were analysed using SPSS for Windows, version 23 (SPSS Inc, IBM Corp, Armonk, NY, USA) and R version 4.0.1 (R Foundation for Statistical Computing, Vienna, Austria).

## RESULTS

### Distribution of right heart failure stages

**Figure 3:**
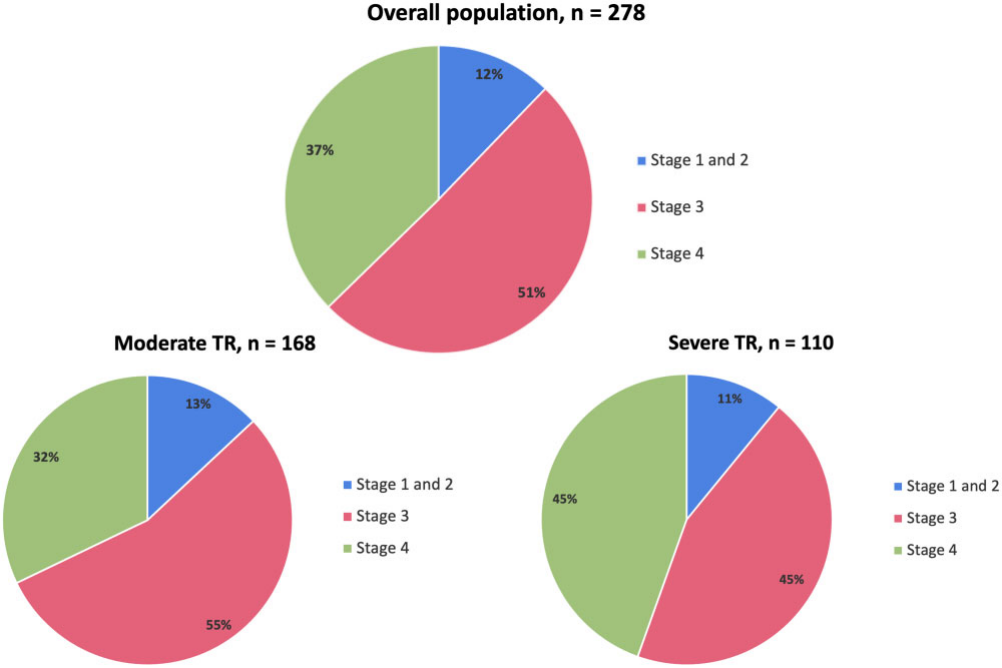
Distribution of patients with significant tricuspid regurgitation across stages of right heart failure for the overall population and according to the severity of tricuspid regurgitation. TR: Tricuspid regurgitation.

A total of 278 patients [64 (12) years, 49% males] diagnosed with significant TR, who subsequently underwent TV surgery were included in the analysis. At the time of first diagnosis of significant TR on echocardiography, 34 patients (12%) were in stages 1 and 2, 141 patients (51%) were in stage 3 and 103 patients (37%) were in stage 4 (Fig. 2). Moderate TR was diagnosed in 168 patients (60%) and severe TR in 110 patients (40%), with an equal distribution of TR severity over the different right heart failure stages (severe TR was diagnosed in 11%, 45% and 45%, respectively in stages 1 and 2, stage 3 and stage 4; *P* = 0.11; Fig. 3).

### Clinical characteristics

Clinical characteristics of the overall population stratified according to the different right heart failure stages are presented in Table 1. Of the 278 patients who had TV surgery, 265 patients (95%) underwent TV annuloplasty, 9 patients (3%) had TV replacement with a bioprosthetic valve and 4 patients (1%) had TV replacement with a mechanical prosthesis. The majority of patients (91%) had TV surgery concomitant to left-sided valve surgery or coronary artery bypass grafting.

**Table 1:** Baseline characteristics of the total population and according to stages of right heart failure

Characteristics	Overall population (*n* = 278)	Stages 1 and 2 (*n* = 34)	Stage 3 (*n* = 141)	Stage 4 (*n* = 103)	*P*-Value	*P*-Value for trend analysis
Demographic characteristics						
Age, years	64(12)	67(10)	63(13)	66(10)	0.10	0.72
Male sex	135(49)	14(41)	64(45)	57(55)	0.20	0.083
Body surface area, m^2^	1.9(0.2)	1.9(0.2)^c^	1.9(0.2)^c^	2.0(0.2)^a,b^	0.008	0.002
Medical history						
NYHA functional class					<0.001	<0.001
I	53(19)	29(100)^bc^	17(12)^a^	7(7)^a^		<0.001
II	66(24)	0(0)^b^	51(36)^ac^	15(15)^b^		0.53
III	111(41)	0(0)^bc^	73(52)^a^	38(37)^a^		0.13
IV	43(16)	0(0)^c^	0(0)^c^	43(42)^ab^		<0.001
Oedema	77(28)	0(0)^c^	0(0)^c^	77(75)^ab^	<0.001	<0.001
Hypertension	197(73)	21(68)	100(73)	76(76)	0.64	0.35
Dyslipidaemia	107(40)	9(29)	55(40)	43(43)	0.38	0.21
Diabetes mellitus	42(16)	1(3)^c^	16(12)^c^	25(25)^ab^	0.002	0.001
(Ex-)smoker	99(37)	10(32)	43(31)	46(47)	0.05	0.035
Coronary artery disease	86(31)	6(19)	40(29)	40(39)	0.06	0.018
Pacemaker/ICD	85(31)	9(27)	41(29)	35(34)	0.61	0.33
Atrial fibrillation	153(56)	18(56)	75(54)	60(59)	0.70	0.55
COPD	38(14)	2(6)	17(12)	19(18)	0.33	0.70
Laboratory values						
Haemoglobin, mmol/l	7.9(1.3)	7.8(1.5)	7.9(1.3)	7.8(1.3)	0.78	0.78
Creatinine, µmol/l	92(73–121)	77(64–91)^c^	92(71–120)	103(79–130)^a^	0.002	0.067
Hepatic congestion	153(60)	11(39)^c^	68(52)^c^	74(76)^a,b^	<0.001	<0.001
Medication						
Beta-blocker	161(61)	15(52)	89(66)	57(58)	0.23	0.87
ACE-inh/ARB	168(64)	14(48)	93(69)	61(62)	0.09	0.65
MRA	80(31)	2(7)^bc^	42(31)^a^	36(37)^a^	0.009	0.007
Loop diuretic	170(62)	0(0)^bc^	91(65)^a^	79(77)^a^	<0.001	<0.001
Risk scores						
EuroScore II	2.6(1.6–4.6)	1.8(1.2–2.4)^c^	2.4(1.3–4.0)^c^	3.4(1.8–6.6)^a,b^	<0.001	0.001
Charlson Comorbidity Index	4(2)	3(1)	4(2)	5(2)	<0.001	<0.001
Tri-score	3(2–5)	2(1–2)^b,c^	3(2–4)^a,c^	6(4–7)	<0.001	<0.001
Surgical characteristics						
Type of TV intervention					0.26	0.49
TV annuloplasty	265(95)	32(94)	134(95)	99(96)		0.60
TVR bioprosthetic valve	9(3)	2(6)	3(2)	4(4)		0.90
TVR mechanical prosthetic valve	4(1)	0(0)	4(3)	0(0)		0.45
Concomitant surgery	253(91)	32(94)	124(88)	97(94)	0.19	0.48
Concomitant mitral valve surgery	212(76)	25(74)	115(82)	72(70)	0.10	0.23
Time diagnosis surgery, months	3(0–9)	5(1–40)^c^	4(1–13)^c^	2(0–7)^ab^	0.003	<0.001

Values are mean(SD), median(IQR) or *n*(%). Bonferroni correction: ^a^*P* < 0.05 versus stages 1 and 2; ^b^*P* < 0.05 versus stage 3; and ^c^*P* < 0.05 versus stage 4. Hepatic congestion was assessed by the presence of elevated alkaline phosphatase or gamma glutamyl transferase, according to the laboratory reference values.

ACE-inh: angiotensin-converting enzyme inhibitor; ARB: angiotensin receptor blockers; COPD: chronic obstructive pulmonary disease; ICD: implantable cardioverter–defibrillator; IQR: interquartile range; MRA: mineralocorticoid receptor antagonist; NYHA: New York Heart Association; TV: tricuspid valve; TVR: tricuspid valve replacement.

No significant differences in age or sex were observed across the right heart failure stages. Patients with more advanced stages of right heart failure presented with worse renal function and more prominent hepatic dysfunction, assessed by the presence of elevated alkaline phosphatase or gamma-glutamyl transferase, according to the laboratory reference values. Inherent to the classification system of right heart failure used in this study, significant differences among the stages were observed in New York Heart Association (NYHA) functional class, peripheral oedema and diuretic use.

### Echocardiographic characteristics

The mean left ventricular ejection fraction was 44 (15)%, and concomitant significant aortic stenosis or mitral regurgitation was present in 22% and 54% of patients, respectively (Table 2).

**Table 2:** Echocardiographic characteristics of the total population and according to stages of right heart failure

Characteristics	Overall population (*n* = 278)	Stages 1 and 2 (*n* = 34)	Stage 3 (*n* = 141)	Stage 4 (*n* = 103)	*P*-Value	*P*-Value for trend analysis
LV, LA, and left-sided valvular disease						
LV end-diastolic diameter, mm	53(11)	48(10)^c^	51(11)^c^	56(12)^ab^	<0.001	<0.001
LV ejection fraction, %	44(15)	50(12)^c^	46(15)^c^	40(15)^ab^	<0.001	<0.001
LA end-systolic volume—indexed, ml/m^2^	55(39–75)	45(29–66)	55(38–74)	56(42–76)	0.13	0.33
E/A ratio	1.9(1.1–3.0)	1.3(0.9–1.8)^c^	1.9(1.1–3.2)	2.2(1.5–3.2)^a^	0.007	0.014
Moderate/severe aortic stenosis	59(22)	4(13)	29(21)	26(26)	0.29	0.12
Moderate/severe mitral regurgitation	148(54)	13(38)	76(55)	59(57)	0.15	0.10
RV and RA						
RV basal dimension, mm	48(10)	46(9)	48(10)	50(9)	0.13	0.042
RV end-diastolic area, mm^2^	26(10)	23(8)	25(9)	28(10)	0.04	0.012
RV fractional area change, %	35(12)	39(12)	35(12)	34(12)	0.15	0.098
TAPSE, mm	17(5)	18(6)^c^	18(5)^c^	15(5)^ab^	0.002	0.001
RV systolic pressure, mmHg	46(16)	42(13)	46(17)	48(17)	0.28	0.13
RA maximum area, mm^2^	27(21–34)	26(20–36)	26(21–33)	28(24–35)	0.10	0.16
Tricuspid valve						
Severe tricuspid regurgitation	110(40)	12(35)	49(35)	49(48)	0.11	0.071
Valvular annulus diameter, mm	43(8)	41(7)	42(8)	44(8)	0.13	0.042
Vena contracta, mm	9.3(6.9–13.0)	8.0(6.4–10.7)	9.0(6.5–13.0)	10.0(7.7–13.0)	0.09	0.053
EROA, mm^2^	55.8(36.4–95.1)	44.8(37.3–58.8)	57.4(33.0–90.0)	61.3(38.3–110.1)	0.17	0.36

Values are mean(SD), median(IQR) or *n*(%). Bonferroni correction: ^a^*P* < 0.05 versus stages 1 and 2; ^b^*P* < 0.05 versus stage 3; and ^c^*P* < 0.05 versus stage 4.

EROA: effective regurgitant orifice area; LA: left atrial; LV: left ventricle; RA: right atrial; RV: right ventricle; TAPSE: tricuspid annular plane systolic excursion.

In the per-group analysis, patients in stage 4 of right heart failure had significantly larger left ventricular and RV dimensions, lower left ventricular ejection fraction and more severe diastolic dysfunction as compared to patients with other right heart failure stages. Inherent to the classification system of right heart failure used in this study, significant differences across the stages were observed in TAPSE (additional analysis according to RV dysfunction is provided in Supplementary Material, Table S2 and Supplementary Material, Fig. S2).

### Peri- and postoperative morbidity and mortality

No significant differences in valve deterioration or morbidity (thrombus, embolism, bleeding, operated valve endocarditis, reintervention or need for new pacemaker/implantable cardioverter defibrillator within 14 days after surgery) were found across the different stages of right heart failure (Supplementary Material, Table S3).

### Prognostic impact of right heart failure staging

**Figure 4:**
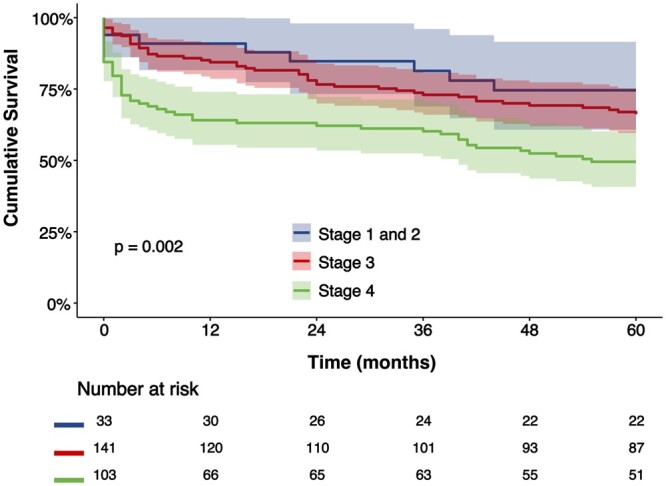
Kaplan–Meier curves for overall survival according to stages of right heart failure. The curves demonstrate the Kaplan–Meier curves for overall survival, respectively for stages 1 and 2, stage 3 and stage 4; with overlaid 95% confidence intervals—shaded areas.

During a median follow-up of 75 (IQR: 19–114) months and a median follow-up index of 0.75 (IQR: 0.16–1.00), after TV surgery was performed, 147 deaths (53%) occurred. The cumulative survival rates were 89%, 78% and 61% at 1 month, 1 year and 5 years, respectively. The Kaplan–Meier curves for overall survival according to the different right heart failure stages are shown in Fig. 4. Survival rates at the 5-year follow-up were significantly worse in patients with more advanced stages of right heart failure: 75%, 66% and 50% for stages 1 and 2, stage 3 and stage 4, respectively (log-rank chi-square: 12.160; *P* = 0.002).

Univariable and multivariable Cox regression analyses for all-cause mortality are presented in Table 3 (and additional sensitivity analyses are available in Supplementary Material, Table S4). The overall staging model of right heart failure, adjusted for clinically relevant confounding factors, was significantly associated with all-cause mortality. Moreover, both ‘stages 1 and 2’, as well as ‘stage 3’ had significantly better hazards for survival compared to ‘stage 4’.

**Table 3:** Univariable and multivariable Cox proportional hazard models for all-cause mortality in patients with significant tricuspid regurgitation who underwent tricuspid valve surgery

Variable	Univariable analysis		Multivariable analysis	
	Hazard ratio (95% CI)	*P*-Value	Hazard ratio (95% CI)	*P*-Value
Age, years	1.009 (0.992–1.025)	0.32	1.019 (0.999–1.039)	0.062
Male sex	1.446 (0.987–2.116)	0.058	0.983 (0.627–1.542)	0.94
Body surface area, m^2^	1.295 (0.517–3.246)	0.58		
NYHA functional class		0.061		
NYHA I (reference)	Ref.	Ref.		
NYHA II	0.813 (0.419–1.578)	0.54		
NYHA III	1.481 (0.854–2.568)	0.16		
NYHA IV	1.695 (0.888–3.237)	0.11		
Oedema	1.436 (1.089–1.893)	0.010		
Arterial hypertension	0.852 (0.557–1.303)	0.46		
Dyslipidaemia	1.532 (1.041–2.256)	0.031		
Diabetes mellitus	1.757 (1.112–2.777)	0.016		
(Ex-)smoker	1.281 (0.865–1.899)	0.22		
Coronary artery disease	1.661 (1.125–2.452)	0.011		
Pacemaker-/ICD-lead	2.429 (1.661–3.555)	<0.001	2.164 (1.400–3.344)	0.001
Atrial fibrillation	0.839 (0.572–1.230)	0.37		
Haemoglobin, mmol/l	0.934 (0.807–1.082)	0.36	0.954 (0.808–1.126)	0.58
Creatinine, µmol/l	1.005 (1.003–1.007)	<0.001	1.005 (1.003–1.007)	<0.001
Hepatic congestion	1.715 (1.130–2.604)	0.011	1.153 (0.725–1.835)	0.55
Loop diuretic	1.873 (1.222–2.872)	0.004		
Time diagnosis surgery, days	1.000 (1.000–1.000)	0.443	1.000 (0.999–1.000)	0.55
Concomitant mitral valve surgery	0.601 (0.397–0.909)	0.016	0.589 (0.371–0.937)	0.025
LVEF, %	0.977 (0.965–0.990)	<0.001	0.990 (0.975–1.005)	0.19
Left atrial end-systolic volume—indexed, ml/m^2^	0.999 (0.994–1.004)	0.71		
Moderate/severe aortic stenosis	1.453 (0.943–2.238)	0.090		
Moderate/severe mitral regurgitation	0.846 (0.579–1.236)	0.39		
RV fractional area change, %	0.986 (0.971–1.001)	0.064		
TAPSE, mm	0.947 (0.908–0.988)	0.011		
RV systolic pressure, mmHg	1.001 (0.989–1.013)	0.89		
RA maximum area, mm^2^	1.007 (0.997–1.023)	0.41		
Severe tricuspid regurgitation	1.396 (0.954–2.042)	0.086		
Valvular annulus diameter, mm	1.017 (0.992–1.042)	0.18	0.988 (0.960–1.018)	0.44
Stages of right heart failure		0.002		0.043
Stages 1 and 2	0.391 (0.186–0.823)	0.013	0.383 (0.156–0.942)	0.037
Stage 3	0.548 (0.369–0.813)	0.003	0.636 (0.405–0.997)	0.049
Stage 4 (reference)	Ref.	Ref.	Ref.	Ref.

Hepatic congestion was assessed by the presence of elevated alkaline phosphatase or gamma glutamyl transferase, according to the laboratory reference values.

CI: confidence interval; ICD: implantable cardioverter–defibrillator; LVEF: left ventricular ejection fraction; NYHA: New York Heart Association; RA: right atrium; RV: right ventricle; TAPSE: tricuspid annular plane systolic excursion.

## DISCUSSION

The aim of the present study was whether a staging algorithm of right heart failure is associated with outcomes, analysed individually as well as corrected for variables that have been associated with outcome in valvular heart disease; and not to build a prediction model. The main findings of the present study are two-fold: (i) the majority of patients with significant TR are referred for surgical intervention at a late stage, with symptoms and signs of right heart failure, and (ii) stages 1 and 2 and stage 3 of right heart failure are associated with better outcomes after surgery compared to stage 4.

### Tricuspid valve surgery and right heart failure

Timing of surgical intervention for severe TR remains challenging. Current guidelines recommend (preferably) surgical TV repair in patients with symptomatic severe TR and in patients with a dilated TV annulus who undergo left-sided valve surgical intervention [3, 4]. However, severe TR may remain asymptomatic for a long time and when symptoms are present, diuretic treatment effectively improves the symptoms, leading to a low referral rate for intervention. Nevertheless, data from various registries have shown poor survival when severe TR is left untreated (without intervention) [1, 2]. However, recent series reporting on the outcomes of (isolated) TV surgical intervention for severe TR have also shown relatively high in-hospital mortality rates [22–24]. An analysis of 54 375 patients from The Society of Thoracic Surgeons Database undergoing TV surgery revealed a small decrease in operative mortality for TV surgery from 10.6% in 2000 to 8.2% in 2010 (*P* < 0.001), despite a higher comorbidity burden with time; however, mortality rates remained higher when compared to those of other valve interventions. Congestive heart failure with NYHA functional class I–III symptoms as well as congestive heart failure with NYHA functional class IV symptoms were independently associated with increased mortality risk, odds ratio: 1.236 (95% confidence interval: 1.135–1.346) and 1.938 (95% confidence interval: 1.764–2.128), respectively [22]. Kim *et al.* showed that among 449 patients with severe TR undergoing TV surgery 47% presented with NYHA functional class III or IV heart failure symptoms. Moreover, NYHA functional class IV was independently associated with an increased risk of all-cause mortality and with the occurrence of the composite end-point of death, TV reoperation and congestive heart failure [25]. Furthermore, Subbotina *et al.* [26] investigated the influence of preoperative RV function in 191 patients undergoing TV surgery. A total of 83 patients (43%) had reduced RV function preoperatively, of whom 50 patients (61%) presented with NYHA functional class III or IV heart failure symptoms. Reduced RV function and a higher number of acute heart failure episodes preoperatively were independently associated with 30-day mortality after TV surgery.

### Clinical implications

Considering the relatively high mortality of TV surgery, there is a need for a better selection of patients with severe TR who may benefit from surgical TV intervention. Given that both NYHA functional class heart failure symptoms and RV function are independently associated with outcome after TV surgery, this study evaluated the prognostic value of a staging system, which integrates both variables. This novel staging system proved to be independently associated with outcomes and is easily applicable in clinical practice to guide decision-making regarding (earlier) referral for TV surgery.

Transcatheter TV interventions, which are considered less invasive than surgery and have been proven safe and effective in reducing TR, provide a potential future therapeutic option for high-risk patients who were previously deemed inoperable [27–30]. Albeit patients treated with transcatheter TV interventions are older and have more comorbidities compared to patients treated surgically, the 6-month mortality rate of transcatheter interventions (varying from 5% to 10%) appears to be comparable or even moderately better than mortality in TV surgery (ranging around 10%) [22, 23, 27, 28]. Patients with right heart failure stage 3 or 4 who underwent TV surgery experienced a considerably higher 6-month mortality risk compared to patients treated percutaneously [27–30]. The novel staging algorithm evaluating RV dysfunction and signs of right heart failure may help identifying patients who may benefit more from less invasive TV interventions.

### Limitations

First, this study is limited by its retrospective design from a single tertiary centre with a limited number of patients and the results need to be confirmed in larger, prospective cohorts. Second, it is important to acknowledge the limitations in the evaluation of the staging system of RV dysfunction and right heart failure due to the complex RV geometry. Third, symptoms of right heart failure are subjective and other signs of right heart failure (hepatic congestion, ascites, jugular venous distention) might be incremental in the clinical evaluation. Fourth, time from the initial diagnosis of significant TR to TV surgery may significantly influence peri- and postoperative mortality. Accounting for this limitation, the time delay from diagnosis to surgery was included in the multivariable Cox regression analysis. Fifth, the current cohort included a few patients undergoing isolated TV surgery, which did not allow detailed analyses. This could be of interest for future research. Sixth, the design of the study did not allow any prediction statement. Applying the current staging system in future research to develop a prediction model, would be outstanding research. Seventh, clinical follow-up was left at the discretion of the treating physician without systematic clinical follow-up.

## CONCLUSIONS

Patients with significant TR are referred for surgical intervention at a late stage of right heart failure. The survival after surgical TV intervention is better among patients with stages 1 and 2, and stage 3 right heart failure compared to patients in stage 4.

## SUPPLEMENTARY MATERIAL

Supplementary material is available at *EJCTS* online.

## Funding

Jan Stassen received funding from the European Society of Cardiology (ESC Training Grant App000064741). Steele C. Butcher received funding from the European Society of Cardiology (ESC Research Grant App000080404).

**Conflict of interest:** The Department of Cardiology, Heart Lung Centre, Leiden University Medical Centre, has received unrestricted research grants from Abbott Vascular, Bayer, Biotronik, Bioventrix, Boston Scientific, Edwards Lifesciences, GE Healthcare, Medtronic and Novartis. This work was funded by an unrestricted research grant from Edwards Lifesciences (IISUSTHV2018017). Nina Ajmone Marsan received speaker fees from Abbott Vascular and GE Healthcare. Jeroen J. Bax received speaker fees from Abbott Vascular and Edwards Lifesciences. Victoria Delgado received speaker fees from Abbott Vascular, Edwards Lifesciences, GE Healthcare, Medtronic, MSD and Novartis. The remaining authors have nothing to disclose.

## Data Availability Statement

All relevant data are within the manuscript and its supporting information files and are available on reasonable request to the corresponding author.

## Author contributions

**Xavier Galloo:** Conceptualization; Data curation; Formal analysis; Investigation; Methodology; Project administration; Writing—original draft. **Jan Stassen:** Formal analysis; Investigation; Methodology; Writing—review & editing. **Steele C. Butcher:** Conceptualization; Formal analysis; Investigation; Methodology; Validation; Writing—review & editing. **Maria Chiara Meucci:** Formal analysis; Investigation; Methodology; Writing—review & editing. **Marlieke F. Dietz:** Conceptualization; Data curation; Formal analysis; Investigation; Writing—review & editing. **Bart J.A. Mertens:** Conceptualization; Methodology; Resources; Software; Writing—review & editing. **Edgard A. Prihadi:** Formal analysis; Investigation; Methodology; Writing—review & editing. **Pieter van der Bijl:** Formal analysis; Investigation; Visualization; Writing—review & editing. **Nina Ajmone Marsan:** Conceptualization; Data curation; Formal analysis; Funding acquisition; Supervision; Writing—review & editing. **Jerry Braun:** Methodology; Supervision; Visualization; Writing—review & editing. **Jeroen J. Bax:** Conceptualization; Data curation; Project administration; Supervision; Writing—review & editing. **Victoria Delgado:** Conceptualization; Data curation; Formal analysis; Project administration; Supervision; Writing—review & editing.

## Reviewer information

European Journal of Cardio-Thoracic Surgery thanks Francesco Ancona, Michele Di Mauro, David Schibilsky and the other, anonymous reviewer(s) for their contribution to the peer review process of this article.

## Supplementary Material

ezac290_Supplementary_Data

## Notes

Presented at the 2021 ESC Congress: The Digital Experience; Session on New Insights into Cardiovascular Surgery, 28 August 2021.

## ABBREVIATIONS

IQR: Interquartile range
NYHA: New York Heart Association
RV: Right ventricular
TAPSE: Tricuspid annular plane systolic excursion
TR: Tricuspid regurgitation
TV: Tricuspid valve
