# Supplementary material for: Staging right heart failure in patients with tricuspid regurgitation undergoing tricuspid surgery
Source: Eur J Cardiothorac Surg. 2022 May 2;62(2):ezac290. doi: 10.1093/ejcts/ezac290 (PMC9789688; doi:10.1093/ejcts/ezac290)
Supplement: ezac290_Supplementary_Data [file ezac290_supplementary_data.docx]

**Supplementary material**

**Table S1.** Tri-Score as an ordinal variable with the number (percentage) of patients per score value.

|  | **Overall population**  **(n=278)** | **Stage 1 and 2**  **(n=34)** | **Stage 3**  **(n=141)** | **Stage 4**  **(n=103)** | **p-value** |
| --- | --- | --- | --- | --- | --- |
| TriScore*  0  1  2  3  4  5  6  7  8  9  10  11  12 | 3 (2 - 5)  9 (3)  35 (13)  47 (17)  56 (20)  32 (12)  30 (11)  25 (9)  29 (10)  3 (1)  10 (4)  1 (0)  0 (0)  1 (0) | 2 (1 - 2)^b,c^  3 (9)  11 (32)  12 (35)  4 (12)  1 (3)  2 (6)  1 (3)  0 (0)  0 (0)  0 (0)  0 (0)  0 (0)  0 (0) | 3 (2 – 4)^a,c^  6 (4)  23 (16)  30 (21)  40 (28)  14 (10)  12 (9)  8 (6)  6 (4)  0 (0)  2 (1)  0 (0)  0 (0)  0 (0) | 6 (4 – 7)^a,b^  0 (0)  1 (1)  5 (5)  12 (12)  17 (17)  16 (16)  16 (16)  23 (22)  3 (3)  8 (8)  1 (1)  0 (0)  1 (1) | <0.001 |

Values are median (IQR) or n (%). ^a^p<0.05 vs stage 1 and 2; ^b^p<0.05 vs stage 3; ^c^p<0.05 vs stage 4.

*Tri-Score analysed as a continuous variable as well as ordinal variable with the number (percentage) of patients per score value.

**Table S2.** RV dysfunction according to TAPSE and RV FAC, assessed in the overall population and per stage of right heart failure

|  | **Overall population**  **(n=278)** | **Stage 1 and 2**  **(n=34)** | **Stage 3**  **(n=141)** | **Stage 4**  **(n=103)** | **p-value** |  |
| --- | --- | --- | --- | --- | --- | --- |
| **TAPSE** | | | | | | |
| TAPSE <17 mm | 140 (54) | 14 (42) | 61 (47) | 65 (66)^a,b^ | 0.006 |  |
| TAPSE <15 mm | 93 (36) | 9 (27) | 34 (26) | 50 (51)^b^ | <0.001 |  |
| TAPSE <13 mm | 49 (19) | 4 (12) | 18 (14) | 27 (28)^b^ | 0.019 |  |
| **RV FAC** | | | | | | |
| RV FAC <30% | 94 (34) | 4 (12) | 53 (38)^a^ | 37 (37)^a^ | 0.017 |  |
| RV FAC <25% | 64 (23) | 3 (9) | 33 (23) | 28 (28) | 0.089 |  |
| RV FAC <20% | 31 (11) | 3 (3) | 17 (12) | 11 (11) | 0.879 |  |

Values are n (%). ^a^p<0.05 vs stage 1 and 2; ^b^p<0.05 vs stage 3; ^c^p<0.05 vs stage 4.

RV FAC, right ventricular fractional area change; TAPSE, tricuspid annular plane systolic excursion.

**Table S3.** Peri- and postoperative morbidity and mortality

|  | **Overall population**  **(n=278)** | **Stage 1 and 2**  **(n=34)** | **Stage 3**  **(n=141)** | **Stage 4**  **(n=103)** | **p-value** |
| --- | --- | --- | --- | --- | --- |
| **Mortality** | | | | | |
| All-cause mortality  In-hospital mortality  30-day mortality  90-day mortality | 147 (53)  34 (23)  23 (16)  39 (27) | 13 (38)^c^  3 (23)  2 (15)  2 (15)^c^ | 65 (46)^c^  9 (14)^c^  5 (8)^c^  9 (14)^c^ | 69 (67)^a,b^  22 (32)^b^  16 (23)^b^  28 (41)^a,b^ | 0.001 |
| Cardiovascular death* | 44 (70) | 1 (20) | 16 (76) | 27 (73) | 0.039 |
| Valve related death | 2 (3) | 0 (0) | 1 (5) | 1 (3) | 0.834 |
| **Morbidity** | | | | | |
| Structural valve deterioration | 33 (12) | 4 (12) | 19 (14) | 10 (10) | 0.668 |
| Non-structural valve deterioration | 35 (13) | 1 (3) | 23 (16) | 11 (11) | 0.089 |
| Thrombus | 1 (0) | 0 (0) | 0 (0) | 1 (1) | 0.428 |
| Embolism | 3 (1) | 1 (3) | 1 (1) | 1 (1) | 0.506 |
| Bleeding | 51 (18) | 6 (18) | 28 (20) | 17 (17) | 0.800 |
| Operated valve endocarditis | 8 (3) | 2 (6) | 2 (1) | 4 (4) | 0.268 |
| Reintervention  Tricuspid valve  Left-sided valve | 17 (6)  6 (35)  11 (65) | 4 (12)  2 (50)  2 (50) | 9 (6)  4 (44)  5 (56) | 4 (4)  0 (0)^a^  4 (100) | 0.325 |
| New PM/ICD within 14d | 12 (4) | 3 (9) | 5 (4) | 4 (4) | 0.356 |

Values are n (%). ^a^p<0.05 vs stage 1 and 2; ^b^p<0.05 vs stage 3; ^c^p<0.05 vs stage 4.

* Cause of death (cardiovascular vs non-cardiovascular) was available in 63 out of 147 patients (43%) in the overall population and for stage 1 and 2, stage 3 and stage 4 in 5 patients (38%), 21 patients (32%) and 37 patients (54%), respectively.

PM, pacemaker; ICD, implantable cardioverter defibrillator.

**Table S4.** Sensitivity analysis for the multivariable cox regression analysis with different models

| **Variable** | **Model 1** | | **Model 2** | | **Model 3** | |
| --- | --- | --- | --- | --- | --- | --- |
|  | **Hazard Ratio (95%CI)** | **p-value** | **Hazard Ratio (95%CI)** | **p-value** | **Hazard Ratio (95%CI)** | **p-value** |
| Age, years | 1.019 (0.999-1.039) | 0.066 | 1.018 (0.998-1.038) | 0.081 | 1.019 (0.999-1.040) | 0.057 |
| Male sex | 1.124 (0.720-1.753) | 0.608 | 1.065 (0.686-1.655) | 0.778 | 1.027 (0.655-1.611) | 0.908 |
| Diabetes mellitus | 0.918 (0.531-1.589) | 0.761 | 1.044 (0.630-1.729) | 0.868 |  |  |
| Chronic obstructive pulmonary disease | 1.092 (0.613-1.948) | 0.765 | 1.034 (0.580-1.842) | 0.910 |  |  |
| Pacemaker-/ICD-lead | 2.072 (1.287-3.338) | 0.003 | 1.951 (1.246-3.055) | 0.003 | 2.331 (1.485-3.657) | <0.001 |
| Creatinine, µmol/L | 1.004 (1.002-1.007) | <0.001 | 1.005 (1.003-1.007) | <0.001 | 1.005 (1.003-1.007) | <0.001 |
| Haemoglobin, mmol/L |  |  |  |  | 0.977 (0.822-1.161) | 0.793 |
| Hepatic dysfunction |  |  | 1.305 (0.824-2.006) | 0.256 |  |  |
| Time diagnosis-surgery, days | 1.000 (1.000-1.000) | 0.579 |  |  | 1.000 (1.000-1.000) | 0.505 |
| Concomitant mitral valve surgery |  |  |  |  | 0.555 (0.344-0.896) | 0.016 |
| Left ventricular ejection fraction, % | 0.990 (0.975-1.005) | 0.190 | 0.992 (0.977-1.007) | 0.302 |  |  |
| Valvular annulus diameter, mm | 0.978 (0.948-1.010) | 0.173 | 0.989 (0.961-1.018) | 0.459 | 0.978 (0.947-1.010) | 0.171 |
| PASP, mmHg | 0.998 (0.985-1.010) | 0.695 |  |  | 0.996 (0.983-1.009) | 0.558 |
| Stages of right heart failure |  | 0.028 |  | 0.039 |  | 0.015 |
| Stage 1 and 2 | 0.344 (0.133-0.886) | 0.027 | 0.361 (0.139-0.940) | 0.037 | 0.342 (0.142-0.822) | 0.016 |
| Stage 3 | 0.629 (0.402-0.985) | 0.043 | 0.633 (0.404-0.992) | 0.046 | 0.613 (0.393-0.955) | 0.030 |
| Stage 4 (Ref.) | Ref. | Ref. | Ref. | Ref. | Ref. | Ref. |

ICD, implantable cardioverter defibrillator; PASP, pulmonary artery systolic pressure.

**Figure S1**. Tri-Score with the number of patients (A) and the percentage per stage of right heart failure (B) per score value.


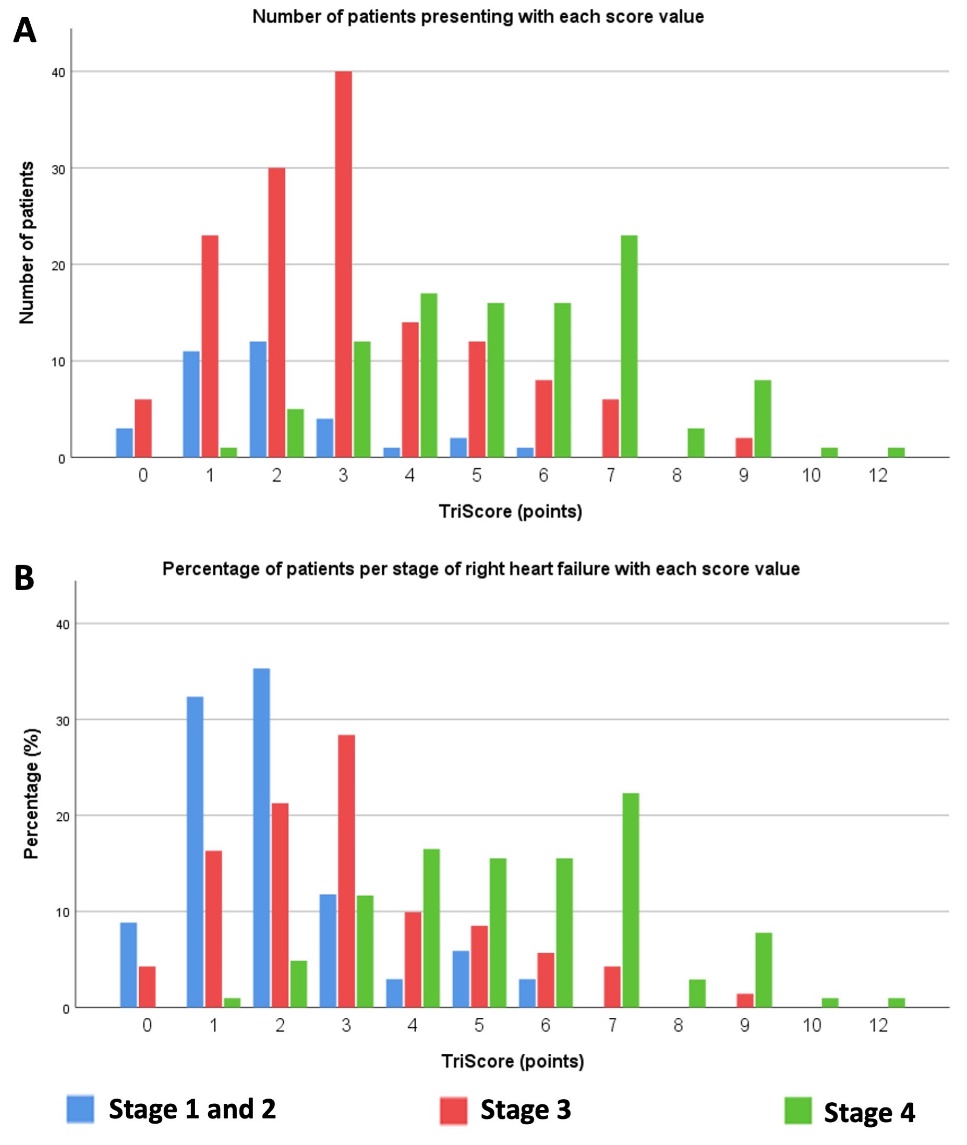


**Figure S2.** Kaplan-Meier curves for overall survival according for the overall population assessed by TAPSE and RV FAC, respectively for mild, moderate and severe RV dysfunction.


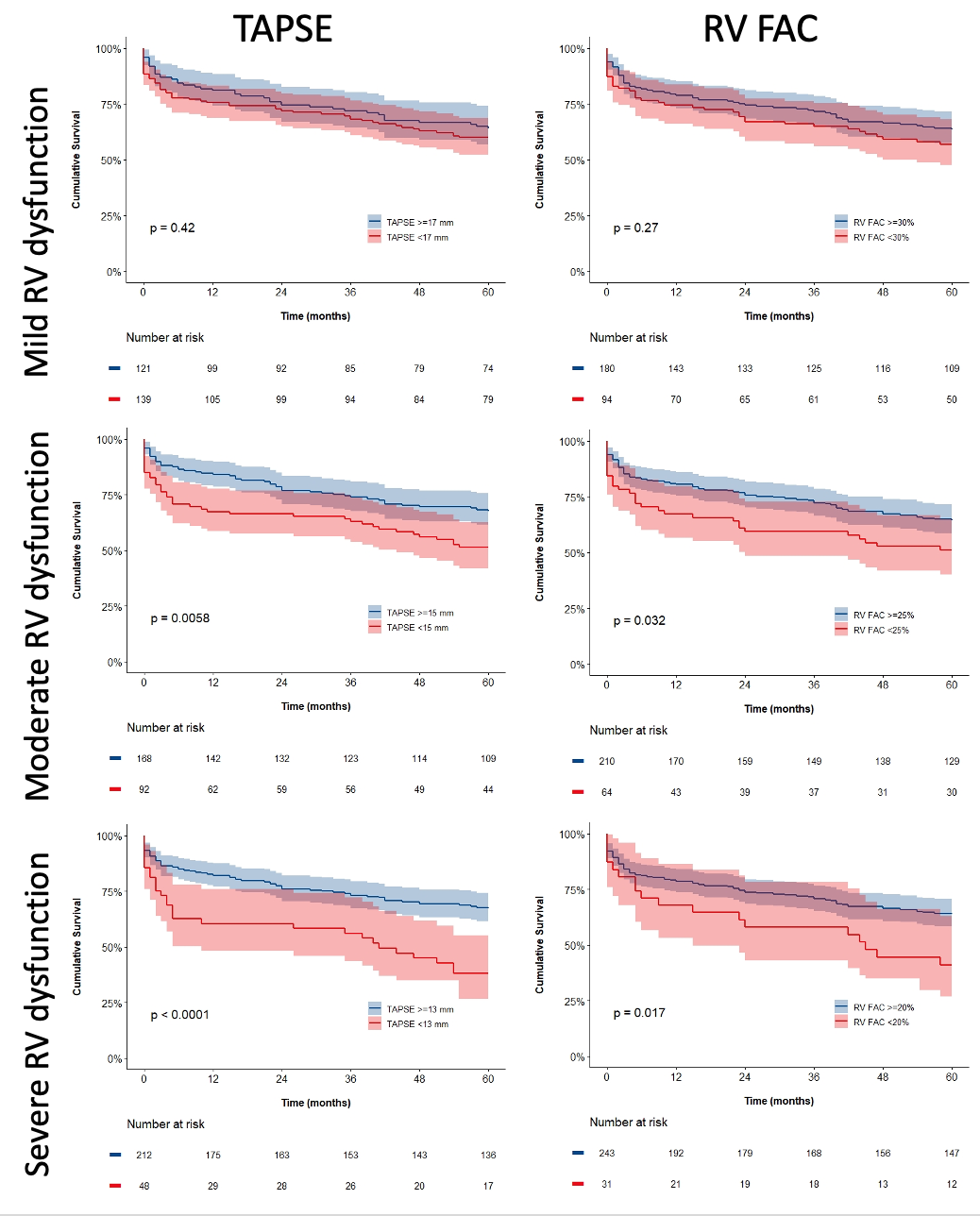


RV, right ventricle; RV FAC, right ventricular fractional area change; TAPSE, tricuspid annular plane systolic excursion.
